# Social Media Use and Mental Health in Adolescents and Young Adults: A Systematic Review and Meta-Analysis of Psychological Distress and Well-Being

**DOI:** 10.1192/j.eurpsy.2026.11144

**Published:** 2026-07-31

**Authors:** Y.-J. Lien, H.-P. Feng

**Affiliations:** 1 Department of Health Promotion and Health Education, National Taiwan Normal University; 2 College of Nursing, National Defense Medical University, Taipei, Taiwan

## Abstract

**Introduction:**

Given the widespread use of social media and growing concerns about adolescent mental health, it is crucial to investigate how different patterns of social media use relate to psychological well-being and distress. Although several meta-analyses have examined this relationship, many have aggregated results without differentiating among specific usage types.

**Objectives:**

This meta-analysis aims to systematically evaluate how different patterns of social media use, such as screen time, problematic use, and types of engagement (active vs. passive), relate to psychological outcomes in adolescents and young adults.

**Methods:**

A systematic search of six electronic databases was conducted for studies published between January 2000 and May 2025. Fifty-nine independent studies met the inclusion criteria, comprising a total of 547,697 participants (54.5% female; mean age = 16.6 years; age range: 12.0–23.3 years).

**Results:**

Meta-regression analyses showed no significant moderating effects of geographic region, gender, or publication year. Random-effects subgroup analyses revealed a strong positive association between problematic social media use and psychological distress (r = 0.39, 95% CI [0.34, 0.44], p < 0.001), and a weaker but significant link between time spent on social media and distress (r = 0.15, 95% CI [0.09, 0.21], p < 0.001). A modest negative association was also found between problematic use and well-being (r = -0.28, 95% CI [-0.51, -0.01], p = 0.04). Other usage patterns showed no significant associations with well-being.

**Conclusions:**

These findings highlight the complex associations between social media use and mental health in adolescents and young adults. Future longitudinal studies using diverse samples and objective behavioral data are essential to clarify these relationships. The results also emphasize the need for targeted interventions to address the adverse effects of problematic social media use in youth populations.

**Disclosure of Interest:**

None Declared

